# An In-Vitro Investigation into the Fracture Resistance of Prefabricated and Custom-Made Zirconia Crowns for Permanent Molars in Children

**DOI:** 10.3390/dj13020064

**Published:** 2025-01-30

**Authors:** Thikrayat Bani-Hani, Rami S. Al-Fodeh, Ahed M. Al-Wahadni, Elham S. Abu-Alhaija, Mahmoud Al-Hakam

**Affiliations:** 1 Division of Paediatric Dentistry, Preventive Dentistry Department, Faculty of Dentistry, Jordan University of Science and Technology, Irbid 22110, Jordan; 2 Department of Prosthodontics, Faculty of Dentistry, Jordan University of Science and Technology, Irbid 22110, Jordan; rsfodeh@just.edu.jo (R.S.A.-F.); ahed@just.edu.jo (A.M.A.-W.); 3 College of Dental Medicine, QU Health, Qatar University, Doha P.O. Box 2713, Qatar; elhama@qu.edu.qa; 4 Prosthodontics, Faculty of Dentistry, Jordan University of Science and Technology, Irbid 22110, Jordan; mhalmasri@gmail.com

**Keywords:** fracture resistance, zirconia crowns, children

## Abstract

**Background:** Recently, the demand for esthetic restorations has grown dramatically and extended into the pediatric population. The prefabricated zirconia crowns (PZCs) and custom-made zirconia crowns (CZCs) are new esthetic options in pediatric dentistry. However, they are still inadequately tested for use in children. Aim: To determine the fracture resistance and failure mode of the PZC in comparison to the CZC. **Materials and Methods:** In this in-vitro study, thirty cobalt-chromium dies were fabricated by scanning the negative replica of a prefabricated lower first permanent molar zirconia crown. CZCs were designed and milled using two different zirconia brands: Ceramill Zolid-FX (FX) and the Highly-Translucent (HT) zirconia. Dies were randomly assigned to receive either a PZC or a CZC (n = 10 in each group). All crowns were cemented on their respective dies using glass ionomer cement. Following artificial aging, all specimens were loaded to failure. Fracture mode analysis was performed. One-way ANOVA and Bonferroni post-hoc test were used for multiple comparisons across the groups. The significant level was set at *p* ≤ 0.05. **Results:** HT zirconia had a significantly higher fracture load compared to other groups (*p* < 0.05). The mean fracture resistance values were: (3087 ± 385) N for HT zirconia, (2633 ± 300) N for PZCs, and (2483 ± 381) N for FX, with no statistically significant difference in fracture strengths between PZCs and FX. **Conclusions:** HT zirconia crowns showed the highest fracture resistance amongst all groups. The fracture loads of tested crowns exceeded the maximum posterior biteforce. When placed in permanent molars, PZC are expected to perform well under masticatory forces in children.

## 1. Introduction

The management of permanent molars with large carious lesions or extensive defects in children has been always a challenge to dentists. Clinicians often face difficulties in providing successful and durable restorations to pediatric patients due to limited cooperation and limited treatment options.

The preformed metal crowns have been considered the gold standard for restoring extensively carious molars and molars with developmental anomalies in children. For many years, these metal crowns, also known as stainless steel crowns, have offered a safe, effective, and economical restorative option [1]. Despite demonstrating durability and long-term success [2], these crowns are nowadays frequently refused by patients as they lack esthetic appeal. Generally, the demand for esthetic restorations has grown dramatically and become an important factor influencing clinical decisions even in pediatric dentistry [3].

In view of this demand, esthetic alternatives have emerged for use in primary and permanent molars in children [4]. Recently, prefabricated zirconia crowns (PZCs) have been introduced into pediatric dentistry as an esthetic restorative option. Zirconia, which is a polycrystalline ceramic without glass components, provides an esthetic option with favorable mechanical properties, high comprehensive strength (2000 MPa), high flexural strength (900–1200 MPa), and a fracture toughness of 6–8 MPa [5]. These crowns have gained remarkable popularity and high parental satisfaction particularly due to their excellent esthetics and color stability [6]. However, as they are rigid and cannot be adjusted or crimped to fit the tooth, the tooth structure has to undergo a significant preparation to ensure passive fitting of these crowns. Therefore, the clinical adaptation of PZCs is often time-consuming and requires cooperation from the patient [7]. Conversely, over the last decade, PZCs have demonstrated favorable mechanical properties, biocompatibility, and durability in the primary dentition [8,9].

More recently, these prefabricated crowns have been used for restoration of permanent molars in children. However, the question as to whether these crowns can be considered a definitive restorative option remains unanswered. A crown’s durability is strongly related to its mechanical properties. The latter determine a material’s behavior or a reaction under applied external forces [10]. The fracture resistance is an important property that can dictate the behavior of all-ceramic dental materials upon loading. Even if fracture is not considered the main reason for restorative failure in children [11], it is a common cause of replacement of all-ceramic crowns in the posterior area of the mouth [12].

With the advances in digital dentistry and CAD/CAM technologies, a wide range of esthetic restorations have become available for intra-oral application. The CAD/CAM zirconia is an innovative alternative that can be used for restoring primary and permanent molars in children. It is expected that milled crowns may have superior mechanical properties [13] and exhibit better adaptation to the prepared tooth [14]. Furthermore, the milling of crowns by CAD/CAM can significantly reduce the total cost (by approximately 50%) to clinicians in the world where commercial zirconia crowns may be overly expensive or even unavailable [15].

However, the placement of custom-made crowns often requires lab work and cannot be completed in one visit, making them less suitable for use in children.

The literature is rich on studies investigating the mechanical properties of the various restorative options in children [16,17,18]; however, the mechanical properties of PZCs in comparison to the custom-made crowns are still scarcely investigated particularly with regards to their use in permanent molars in children. It is still unknown if the prefabricated crowns should be replaced by custom-made restorations at a certain point when they are placed in permanent molars in children. An insightful understanding of a material’s survival and longevity can be obtained from investigating the fracture strength following a process of artificial aging. Therefore, the aim of the current in-vitro study was to evaluate the fracture resistance of PZCs in comparison to CZCs, made of two commercially available zirconia brands, as well as to record their failure mode. The null hypothesis assumed no significant difference in fracture resistance between the three test groups in this study.

## 2. Materials and Methods

The sample size calculations were performed with the G* power 3.1 program for Macintosh (Heinrich Heine, Universitat Dusseldorf, Dusseldorf, Germany) using data from a previous study [19] (effect size = 1.66, Alpha = 0.05, power = 0.95). The minimum-required sample size for each group was nine (n = 9). The sample size was increased by 10% to yield 10 specimens in each test group (total n =30).

To ensure standardization, instead of preparing a dentiform molar tooth, a perfect-fit negative replica was produced by the impression of the interior surface of a PZC (NuSmile, Houston, TX, USA) for a mandibular first permanent molar. The crown form was filled with epoxy resin that was later scanned using a scanbox scanner (Smart Optics, Sensortechnik GmbH, Bochum, Germany) to design cobalt chromium dies using Marterialise Magics software (Materialise NV, Lovaine, Belgium). Thirty cobalt-chromium dies were printed by a trained dental technician using cobalt-chromium dental alloy powder (Mediloy S-Co, Bego, Bremen, Germany), by using a selective laser melting 3D printing machine SLM 280 HL (SLM solutions AG, Lubeck, Germany). Die replicas were then randomly assigned into one of the three experimental groups.

The custom-made zirconia crowns were designed in the dental laboratory using CAD software (Exocad model creator 3.0; Exocad, GmbH, Bochum, Germany). An individual Standard Tessellation Language (STL) milled file was used to construct the crowns with dimensional parameters similar to the prefabricated crowns (1.5 mm thickness occlusally and 1 mm on axial aspects). The crown thickness was measured and verified with a caliper (Ivoclar Vivadent^®^, Schaan, Liechtenstein).

Two different zirconia brands were used: Ceramill Zolid-FX (FX) and Ceramill Highly Translucent (HT) zirconia by (AmannGirrbach, AG, Koblach, Austria). The composition and manufacturers of the three tested materials are listed in Table 1.

All custom-made crowns were inspected for cracks, chipping and other flaws. Crowns were tried on their respective dies to ensure passive fit.

All crowns were cemented on their respective dies using resin-reinforced glass ionomer cement (FUJI PLUS, GC Corporation, Tokyo, Japan) under the same operator’s finger pressure for 4 min according to the manufacturer’s instructions and allowed to set for 24 h. Cemented crowns were stored in distilled water at 37 °C for 24 h followed by thermocycling and mechanical loading.

Artificial aging that simulated 5 years of clinical service [19] was applied. Specimens first underwent 6000 thermal cycles [20] with distilled water at 5 °C/55 °C for 1 min per cycle (a dwell time of 25s and a transfer time of 10s). Cyclic loading was then applied using a dynamic testing machine located at the School of Dentistry at Jordan University of Science and Technology and designed from a similar machine (Chewing simulator cs-4.4. mechantronic, Westerham, Germany) to simulate the physical parameters of masticatory forces. Specimens underwent 1,200,000 cycles of 50 N force at a 1–1.6 Hz frequency applied perpendicularly on the central groove of each crown along the tooth’s long axis.

Following artificial aging, all crowns were inspected for cracks or fractures using magnifying loupes, then they were all loaded until failure in a universal testing machine (WDW-20, Jinan Testing Equipment IE Corporation, Jinan, China). Uniaxial loading until fracture was applied through a steel ball placed in the center of the crowns, with a crosshead speed maintained at 1 mm/min. The maximum load necessary to fracture each specimen was recorded in newtons (N).

Following a load-to-fracture test, all specimens were visually inspected for their fracture mode and broadly categorized according to the fracture pattern into repairable or catastrophic. Scanning electron microscope (SEM) (Quanta FEG 450, FEI, Amsterdam, The Netherlands) micrographs were performed on randomly selected fractured crowns for each group. The specimens were rinsed with 99% ethanol, air dried, then mounted on aluminum stubs by double sided sticky discs of conductive carbon, and finally sputter-coated with ultra-thin coating of gold alloy (Q150R ES sputter coater, Quorum Technologies, Lewes, UK). Examination under microscope was performed at scale bar 50 µm and magnification at a range of 1200–2400× to assess the surface topography of the samples after fracture.

### Statistical Analysis

Descriptive statistics were calculated using SPSS version 26 (IBM Company, Chicago, IL, USA). Data were normally distributed according to the Shapiro–Wilk test with homogenous variance across test groups. One-way ANOVA was used for multiple comparisons across the various groups. Bonferroni post hoc test was performed for the group comparisons. The significant level was set at *p* value ≤ 0.05.

## 3. Results

The means, standard deviations (SDs), and 95% confidence intervals (C.I.s) for fracture resistance values for the different zirconia crowns used in this study are shown in Table 2.

**Figure 1:**
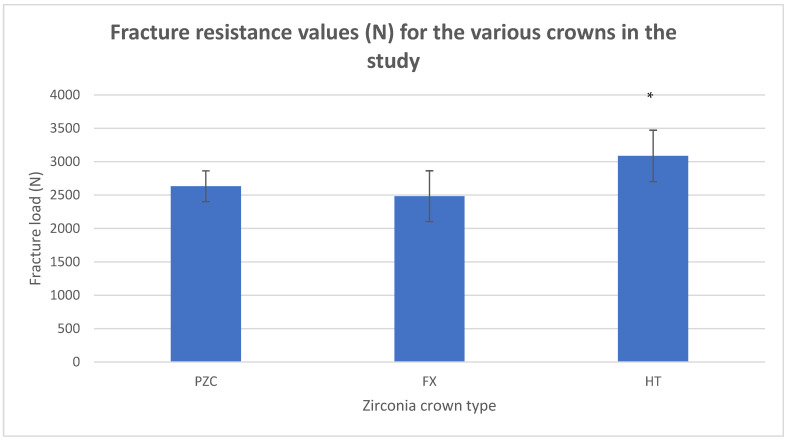
The mean fracture resistance values (N) for the various crowns in the study; PZC: prefabricated zirconia crown (NuSmile, Houston, TX, USA), FX: Ceramill Zolid-FX (FX), and HT: Ceramill Highly Translucent (HT) zirconia by (AmannGirrbach, AG). * Represents a significantly higher fracture load in comparison to other groups (*p*-value < 0.05).

HT zirconia had the highest fracture load, which was significantly higher than the values reported for PZC and FX groups (*p* < 0.05). The results of the study are summarized in Table 2 and Figure 1.

Table 3 shows the results of intergroup comparisons and the significant differences in the fracture resistance as revealed by the post hoc test.

Regarding the fracture mode, all specimens, in the three tested groups, showed a catastrophic mode of fracture, i.e., severe unrepairable type of crown fracture.

**Figure 2:**
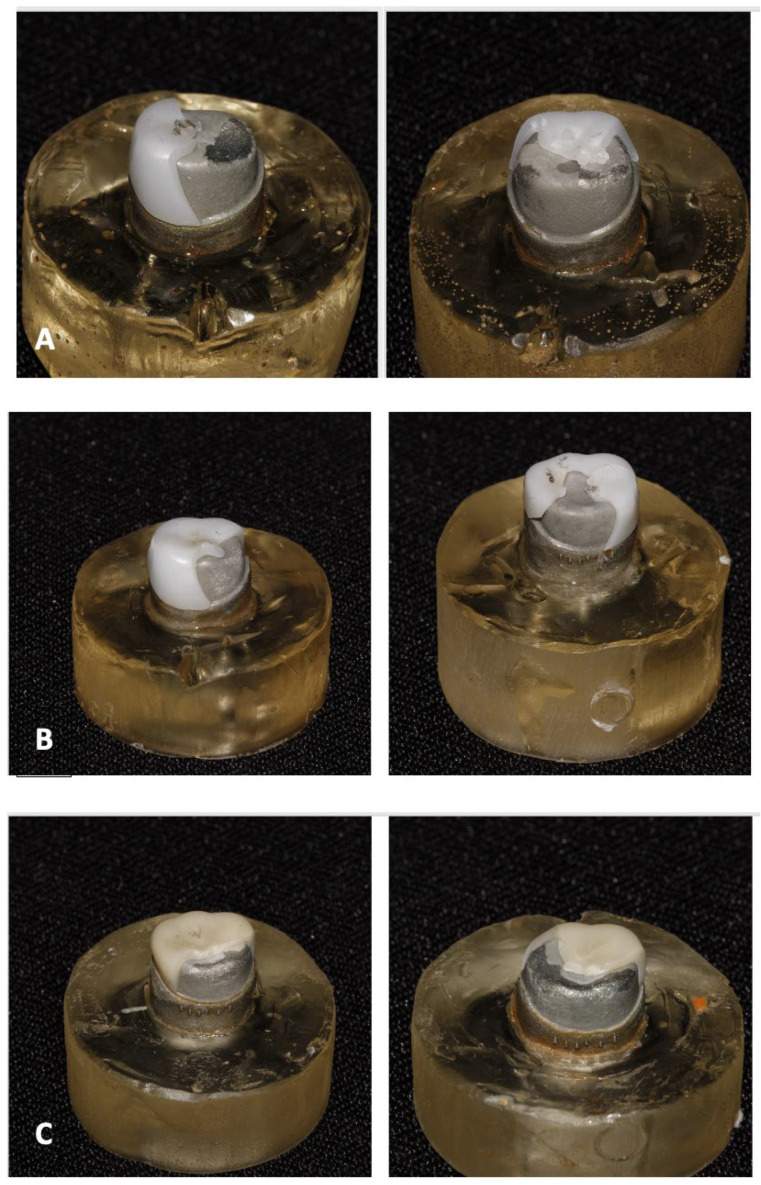
Examples of fractured specimens (**A**): FX, (**B**): HT, (**C**): PZC zirconia crowns.

Figure 2 illustrates some examples of fractured specimens.

**Figure 3:**
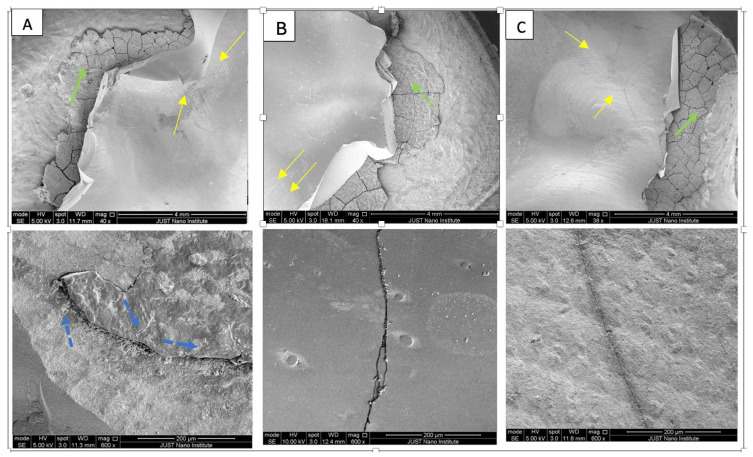
Representative scanning electron microscopy (SEM) micrographs for the fractured samples in each group: (**A**): FX, (**B**): PZC, and (**C**): HT. All tested crowns has catastrophic adhesive failure exposing the cement layer (green arrow). The least extensive fracture was seen with HT zirconia crowns. FX was the most shattered type of crown with radial cracks (blue dashed arrows). Striations or hackle lines (yellow arrows) marking crack propagation direction are illustrated in the figure.

Following SEM analysis, the least extensive fracture was seen with HT zirconia crowns. FX was the most shattered type of crown (Figure 3).

## 4. Discussion

The current study was among the first in the English literature to evaluate the fracture resistance of the prefabricated zirconia crowns in comparison to two commercially available CAD/CAM zirconia brands after subjecting the specimens to an artificial aging process that involves mechanical loading and thermocycling.

The current in-vitro study recorded the highest fracture load for the HT zirconia crowns, followed by the prefabricated crowns. FX crowns showed a lower fracture load. The significant differences and the variation in the fracture resistance of the tested materials in this study could be attributed to their different compositions and microstructure particularly with regards to yttria. The higher yttria content improves the translucency of zirconia; however, it also results in lower fracture resistance [21,22]. The HT zirconia contains 4 mol% yttria as compared to 5 mol% yttria in FX. Based on these results, the null hypothesis was rejected.

In a recent in-vitro investigation, El-Shahawy and Azab compared the fracture resistance of prefabricated versus custom-made zirconia crowns for permanent molars in children. The authors used identical first permeant molar resin dies and measured a mean fracture load of 1793.54 N and 1987.38 N for the PZC (NuSmile, Houston, TX, USA) and custom-made (Cercon ht, Dentsply Sirona, Bensheim, Germany) [23]. Although the type of zirconia (Cercon ht) that was used in the latter study has a lower yttria content (3% mol) in comparison to the zirconia brands in the current research, our experiment reported significantly higher values of fracture load for both prefabricated and milled zirconia crowns. This could be partly explained by the use of metal cobalt-chromium dies in the current study, which have a high Young’s modulus of elasticity resulting in higher fracture loads [19]. It is worth mentioning that the use of cobalt-chromium dies does not mimic the clinical scenario; however, in this study they were used due to their superior strength and stability and this deemed essential for investigating strong and durable materials such as zirconia crowns to ensure that the die does not bend or shatter under strong forces. In another in-vitro study, Elian Al-Hayek et al. (2022) used freshly extracted primary molars and reported a fracture strength of 646.47 N for PZC (NuSmile, Houston, TX, USA) and 2888.60 N for custom-made zirconia crowns (Zirkid) [7]. However, the composition for the milled zirconia brand was not clearly stated. Furthermore, the latter study was conducted on natural and endodontically treated teeth with different caries levels and subsequently different fracture resistance; thus, their findings were incomparable with the results of the current work.

Al-Shobber et al. (2017) studied the fracture resistance of four commercially available esthetic crowns for primary anterior teeth. They reported the highest fracture load (937.36 N) for the PZC (NuSmile, Houston, TX, USA) that were cemented with glass ionomer cement on resin dies [17].

On the other hand, Kist et al. 2019 compared the fracture loads of three brands of prefabricated zirconia crowns, CAD-CAM zirconia crowns, and the conventional stainless steel crowns for primary molars. The authors found that, following artificial aging, the CAD/CAM zirconia crowns had a mean fracture resistance of approximately 2500 N, whereas for PZCs the values ranged from 1700 to 2000 N [19]. In the latter study, the authors concluded that artificial aging (corresponding to 7 years of clinical usage) did not lower the fracture resistance for both the prefabricated and the custom-made zirconia crowns, which concurs with the finding in the present study, i.e., the fracture loads remained adequately high following the aging process.

In the current study, the catastrophic mode was seen in all tested specimens. This might be explained by the high fracture loads as suggested by a previous study [24]. Overall, in the literature, the custom-made zirconia crowns are consistently reported to have a higher fracture resistance compared to the PZCs. A likely explanation is the quality of the marginal fit, which was found to influence the fracture load [19,22]. The custom-made crowns offer other advantages over the prefabricated ones in terms of their relatively easier adaption and fitting in cases of space loss, malocclusion, or malposition [7]. Nonetheless, the prefabricated crowns have been shown to have comparable strength compared to the custom-made restorations. In other words, based on the limited research available so far, there is plausible evidence to anticipate satisfactory performance for the prefabricated crowns for at least up to 5 years [25].

It is worth mentioning that the reported fracture resistance in the literature is not readily comparable and shows great variability due to significant methodological differences. The fracture resistance is influenced by many factors such as crown thickness, composition, luting agent/technique, thickness of cement gap, die material, surface treatment, and others [21], which could also explain the variation across the studies. In this study, all crowns were cemented with GIC as it has been shown that GIC can be used successfully with zirconia crowns [23,26].

In the present study, all fracture loads were far beyond the maximum bite force in children.

Braun et al. (1996) estimated the maximum bite force in young children to be about 78 N for 6-year-olds and 106 n for 10-year-old children [27]. This bite force was found to increase following comprehensive dental treatment in pediatric patients with a mean maximum bite force of 182 N [28]. In another study [29], the occlusal force was found to vary with the type of occlusion and craniofacial dimensions; the bite force in 7-to-13-year old children was estimated to be 349 N in class I, 369 N in class II, and 288 N in class III. In a sample of Jordanian children aged from 4 to 6 years, Owais et al. (2019) evaluated the maximum occlusal bite force (MOBF) changes 6 months after placement of preformed metal crowns (PMCs) on primary molars. In the control group, the mean MOBF at T0 was 140 N and it did not change 6 months later at T5 (141 N). However, in the study group, the mean MOBF values increased from 148 N before the placement of PMCs to 210 N 6 months later [30].

Nonetheless, comparing the fracture loads of the tested materials to the above-mentioned values for the MOBF, it is evident that all tested crowns can well withstand the masticatory forces in children. In the era of esthetic dentistry, pediatric dentists should be encouraged to use new innovative techniques to provide esthetic restorative options for children.

The results of the current investigation should be extrapolated with caution. It has to be acknowledged that artificial replicas that were used in the current experiment are unable to reproduce the actual forces distribution in human teeth that have pulp chambers and root canals. Although the use of natural teeth imposes standardization difficulties as these teeth differ in their age, shapes, sizes, soundness, and storage conditions [22]. However, further studies are recommended with natural teeth or resin dies for better resemblance of real scenarios. Variations in natural teeth can be accounted for by increasing the sample and by employing certain useful statistics. In addition, further research with longer aging periods, and using different types of cements and varying crown thicknesses, are recommended for a better prediction of the long-term performance of these materials.

## 5. Conclusions

The milled HT zirconia showed the highest fracture load among all three groups. The fracture resistance for PZCs was comparable to the custom-made FX, indicating the potential durability of prefabricated pediatric crowns. In comparison to the maximum bite force in the first permanent molar area in children, all materials showed adequate strength. Thus, they all should have sufficient mechanical properties to serve as a medium- or long-term restoration, with at least 5 years of clinical service, in permanent molars in children. Yet, from clinical perspective, the use of prefabricated crowns that can be delivered in the same visit, by trained professionals, is likely more practical in children.

## Figures and Tables

**Table 1:** Composition and manufacturers of the crown materials used in this study.

Crown Brand	Chemical Composition	Manufacturer
Ceramill Zolid-FX white (FX)	ZrO_2_ + HfO_2_ + Y_2_O_3_ ≥ 99%, Y_2_O_3_ 9.15–9.55%, HfO_2_ ≤ 5%, Al_2_O_3_ ≤ 0.5%, other oxides ≤ 1%	Amann Girrbach AG Koblach, Austria
Ceramill Zolid HT (HT)	ZrO_2_ + HfO_2_ + Y_2_O_3_ ≥ 99%, Y_2_O_3_ 6.7–7.2%, HfO_2_ ≤ 5%, Al_2_O_3_ ≤ 0.5%, other oxides ≤ 1%	Amann Girrbach AG Koblach, Austria
NuSmile PZC	ZrO_2_ 88–96% Y_2_O_3_ 4–6% HfO_2_ 5%	NuSmile, Houston, TX, USA

**Table 2:** Comparison of fracture resistance for the different zirconia crowns used in this study.

Group	Mean (N)	Standard Deviation	95% Confidence Interval Lower Upper		ANOVA Test F Value and Significance
PZC (n = 10)	2633	230	2468	2797	8.6 (0.001) ***
FX (n = 10)	2483	381	2211	2755	
HT (n = 10)	3087	385	2811	3362	
Total (n = 30)	2734	419	2578	2891	

*** Significant *p*-value < 0.001.

**Table 3:** Bonferroni post hoc test for correction of multiple comparisons in fracture resistance.

(I) GR	(J) GR	Mean Difference (I–J)	Sig.	95% Confidence Interval	
				Lower Bound	Upper Bound
PZC	FX	149.6	1.000	−238.0158	537.2158
	HT	−453.8 *	0.018 *	−841.4158	−66.1842
FX	PZC	−149.6	1.000	−537.2158	238.0158
	HT	−603.4 *	0.001 *	−991.0158	−215.7842
HT	PZC	453.8 *	0.018 *	66.1842	841.4158
	FX	603.4 *	0.001 *	215.7842	991.0158

* The mean difference is significant at the 0.05 level.

## Data Availability

The data presented in this study are available upon reasonable request from the corresponding author.
